# De-Etiolation of Wheat Seedling Leaves: Cross Talk between Heme Oxygenase/Carbon Monoxide and Nitric Oxide

**DOI:** 10.1371/journal.pone.0081470

**Published:** 2013-12-12

**Authors:** Yahui Liu, Xinna Li, Langlai Xu, Wenbiao Shen

**Affiliations:** 1 Ningbo First Hospital, Ningbo, China; 2 College of Life Sciences, Cooperative Demonstration Laboratory of Centrifuge Technique, Nanjing Agricultural University, State Key Laboratory of Crop Genetics & Germplasm Enhancement, Nanjing Agricultural University, Nanjing, China; 3 Biotechnology Research Institute, Chinese Academy of Agricultural Sciences, Beijing, P.R. China; Temple University, United States of America

## Abstract

Greening of etiolated plants is predominantly stimulated by light but the complete molecular mechanism is still unknown. Multiple studies currently focus on the important physiological effects of heme oxygenase (HO)/carbon monoxide (CO) in plants. In this report, firstly, the role of HO/CO in light-induced de-etiolation process was investigated. We discovered that light could significantly increase HO activities, *HO-1* gene expression, CO release, and chlorophyll accumulation, all of which were sensitive to zinc protoporphyrin (ZnPPIX), the potent inhibitor of HO-1, respectively. Both HO-1 inducer hematin (H) and CO aqueous solution were able to relieve etiolation in wheat seedling leaves under completely darkness by up-regulating endogenous HO/CO system, so as nitric oxide (NO) donor sodium nitroprusside (SNP) did. Similarly, endogenous NO production was also boost in response to light, SNP, hematin and CO aqueous solution in wheat seedling leaves. Additionally, the restoration of chlorophyll contents was blocked, when the inhibitor of mammalian nitric oxide synthase *N^G^*-nitro-L-arginine methylester hydrochloride (L-NAME) or the specific scavenger of NO 2-(4-carboxyphenyl)-4, 4, 5, 5-tetramethylimidazoline-1-oxyl-3-oxide potassium salt (cPTIO) was added, respectively. Furthermore, the inducible effects of light were different from those of SNP, hematin, and CO on Pfr accumulation and *PHYA* transcripts. However, all of sodium nitroprusside (SNP), hematin, and CO could accelerate NO emission, which suggested that HO/CO in wheat seedlings de-etiolation under dark-light transition may have a cross talk with NO.

## Introduction

De-etiolation of etioplasts lack of chlorophyll is a complex developmental process that is triggered predominantly by light. One of the important light-dependent steps is the phototransformation of protochlorophyll (ide) to chlorophyll (ide) [1]. During the subsequent greening period, the rapid accumulation of chlorophylls occurs. In fact, plants have developed elaborate photo sensory systems to optimize its growth and development to daily and seasonal changes. However, the complete molecular mechanisms by which light regulates development are largely unknown.

Heme oxygenases (HOs, EC 1.14.99.3) were originally identified in animals [2], which catalyzes the first and rate-limiting step in the degradation of heme. Via oxidation, HO cleaves heme molecules to yield equimolar quantities of biliverdin IXα (BV), carbon monoxide (CO), and free iron. Indeed, ample evidence currently supports the notion that HO serves to provide potent cytoprotective effects in many models of oxidant-induced cellular and tissue injury both in animals and plants. For example, recent works have indicated the enhancement of HO activities and transcripts in the antioxidant defense system in soybean leaves subjected to lower levels of cadmium (Cd) stress or UV-B irradiation [3], [4].

Moreover, in plants, HOs are not only involved in adaptation or defense against oxidative stress, but also identified to regulate the first step of phycobilin biosynthesis [5]. These linear tetrapyrrole molecules are precursors of the chromophores for the photoreceptor phytochromes, which are traditionally responsible for the detection of far-red light (FR) and red light (R). They are pigments consisting of a polypeptide of about 125 kD carrying a chromophore moiety, the phytochromobilin, which is a linear tetrapyrrole co-valently attached to a conserved cysteine residue in the N-terminal region [6]–[9]. Phytochromes are encoded by gene families in all higher plants [10], each of which uniquely contributes to assemble holo-phytochromes for regulating plant photomorphogenesis.

CO, catalyzed by HOs, was found to play various important physiological roles in animals [11]–[13]. More recently, it has been reported to be involved in various biological processes in plant kingdom. For instance, it acts as a compound with hormonal effects, including affecting the seed dormancy and germination [14], inducing lateral root formation, involving in abscisic acid (ABA)-induced stomatal closure [15], [16], alleviating the cadmium-induced oxidative damage in *Medicago sativa* seedling root tissues [17] and even involving in the auxin-induced cucumber adventitious rooting process [18], most of which were similar to some behaviors reported by NO in plants [19], [20]. Previous reports have shown that NO signal, which was mainly produced by nitric oxide synthase-like protein or nitrate reductase (NR, EC 1.6.6.1/2) in plants [20]–[23], participated in the course of wheat leaves de-etiolation [19]. Considering the similarities between NO and CO, we hypothesized that CO might exert the same function. On the other hand, mounting evidence has demonstrated the presence of CO release in plants [17], [24]–[28], which also provided the evidence for above hypothesis.

To reveal the role of HO/CO system in light-induced de-etiolation, we analyzed the HO expression and CO release of wheat leaves in light-dark transition. In addition, we discussed the mechanism of hematin and CO on restoring the reduction of chlorophyll content in absence of light and suggested a possible link between HO/CO and NO.

## Results

### Light-induced wheat seedlings de-etiolation is sensitive to ZnPPIX, the potent inhibitor of HO-1

**Figure 1:**
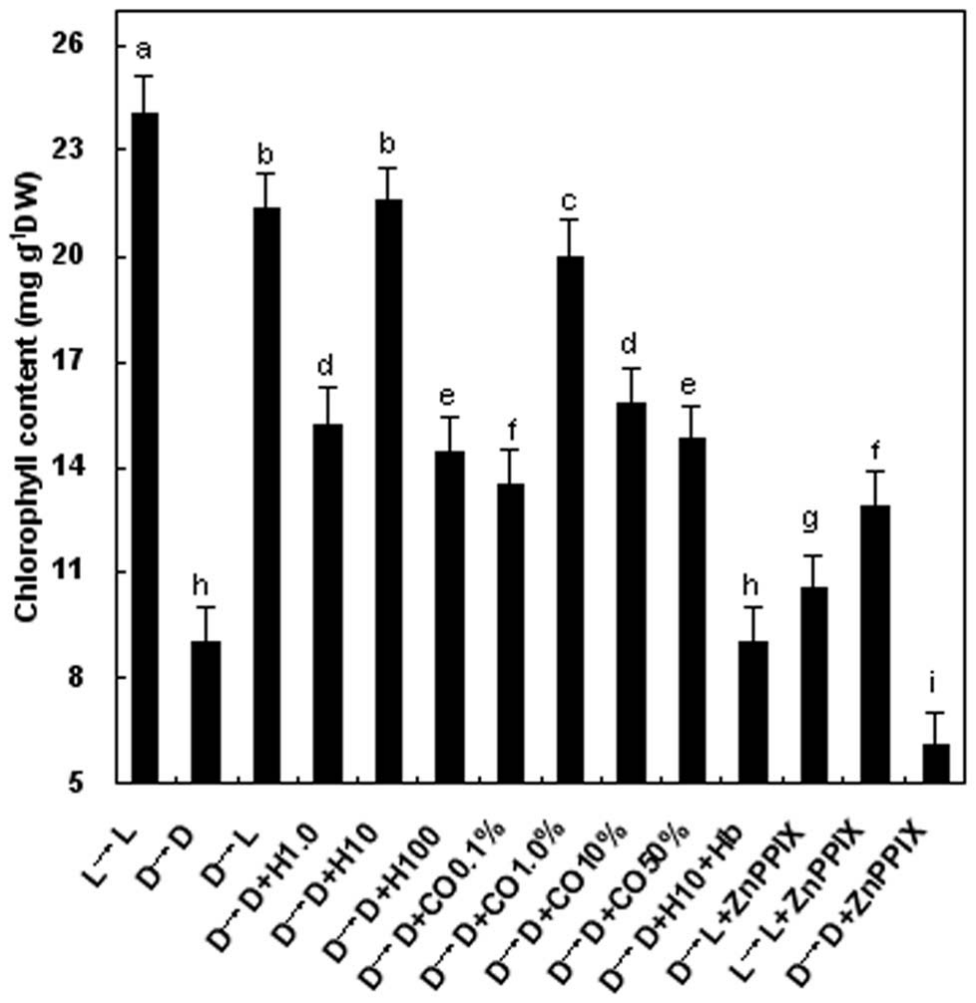
Comparative effects of HO-1 inducer hematin, CO-saturated aqueous solution, and light on the restoration of chlorophyll content in etiolated wheat seedling leaves. 14-day-old wheat seedlings were kept at 25°C in continuous darkness for 5 days. After that, some were transferred into light, while others were still left in darkness without (D→D) or with different contentions of hematin (1.0, 10, and 100 μM, D→D+H1.0, 10, 100), and different saturations of CO aqueous solution (0.1, 1.0, 10, and 50%, D→D+CO0.1, 1.0, 10, 50%). L→L+ZnPPIX, D→D+ZnPPIX, and D→L+ZnPPIX D→H10+Hb stand for combination with HO-1 specific inhibitor ZnPPIX (100 μM), and CO/NO scavenger hemoglobin (Hb, 0.1 gL^−1^), for additional 3 days. L→L stands for wheat seedlings were grown in normal light cycle. Bars denoted by the same letter did not significantly differ at *P*<0.05 according to Duncan's multiple range tests.

In *Arabidopsis*, genetic analysis of the *HY1* locus showed that *hy1* mutant plants display long hypocotyls and decrease chlorophyll accumulation consistent with a substantial deficiency in photochemical active phytochromes (phys) [29]. Meanwhile, the potent HO-1 inhibitor ZnPPIX was first found to inhibit HO activity in both animal and plant tissues [18], [30]. In our test, comparison of the chlorophyll content in seedling leaves of wheat plants transferred from dark to light (D→L) with dark-grown controls (D→D) showed that transferred plants (D→L) maintained relatively higher chlorophyll level after 3 days (*P*<0.05, Fig. 1), as found previously. Interestingly, above light-mediated effect was prevented when ZnPPIX was added (D→L+ZnPPIX), further suggesting a possible interrelationship among light, HO-1, and chlorophyll content in our experimental conditions.

The effects of ZnPPIX on chlorophyll content in the presence of light treatment were also evaluated. As shown in Fig. 1, chlorophyll content of light-grown seedling leaves (L→L) was markedly reduced by addition ZnPPIX (L→L+ZnPPIX, *P*<0.05). Similarly, inhibition of HO-1 activity with ZnPPIX in dark-grown sample resulted in a 33% decrease in chlorophyll content, confirming a possible physiological role for HO-1 in light-induced wheat seedlings de-etiolation.

### Both HO-1 inducer hematin and CO aqueous solution were able to restore the chlorophyll decrease of wheat seedling leaves in completely darkness

**Figure 2:**
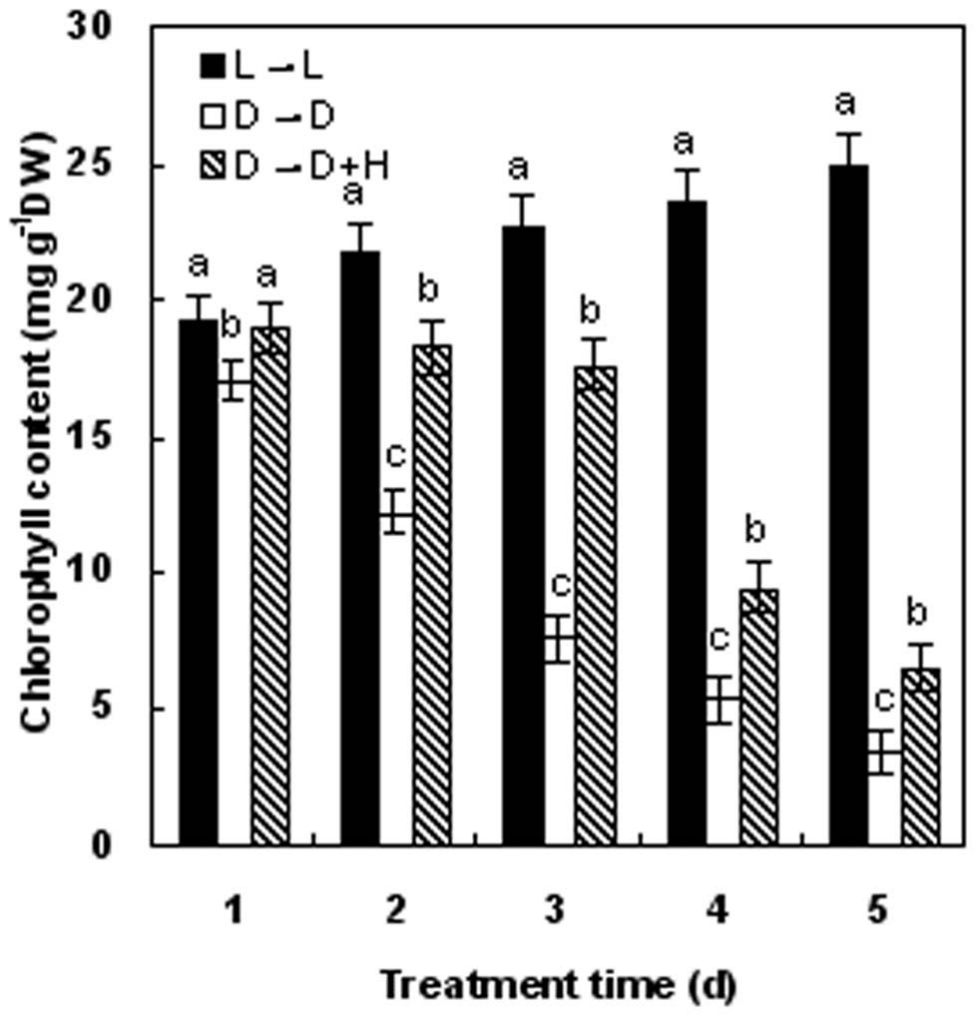
Time course of chlorophyll accumulation following incubation in HO-1 inducer hematin in wheat seedling leaves. Before starting the experiments, 14-day-old wheat seedlings cultured in the Hoagland solution were kept in the light (L, 300 μmmol m^−2^s^−1^) or dark for 5 days. Afterwards, seedlings were cultured in the Hoagland solution with or without 10 μM HO-1 inducer hematin (H) treatment, in the dark (D) for another 5 days. L→L stands for wheat seedlings were grown in normal light cycle. Chlorophyll was extracted and quantified at various times. Bars denoted by the same letter did not significantly differ at *P<*0.05 according to Duncan's multiple range tests.

In further investigations, we noticed that HO-1 inducer hematin (H) or CO aqueous solution (CO), when applied to etiolated wheat seedlings, could dose-dependently restore the loss of chlorophyll content induced by darkness pretreatment. In comparison with dark-grown control plants (D→D), a maximal response at 10 μM hematin and 1.0% CO aqueous solution was observed. Additionally, above hematin-mediated effect was prevented when the CO scavenger hemoglobin (Hb) was added. Thus, above concentration of hematin and CO aqueous solution were used to investigate the role of the HO/CO system throughout this investigation. Furthermore, the results shown in Fig. 2 also confirmed that 10 μM hematin, as an effective inducer of HO-1, was able to partially reverse the negative impact of darkness on chlorophyll content in a time-dependent manner. In a word, above results further illustrated that both hematin and CO aqueous solution could exert the same inducible function as light in continuous darkness.

### Induction of HO/CO system in response to light treatment

**Figure 3:**
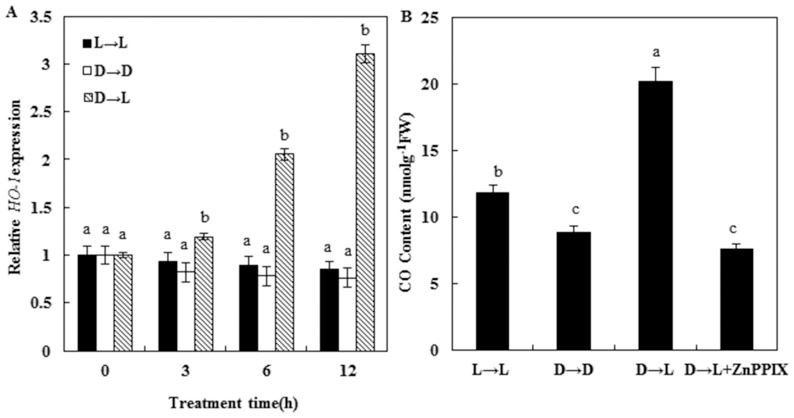
Time course of *HO-1* gene expression in wheat seedling leaves during transition from dark to light for additional hours. A, *HO-1* mRNA expression was analyzed by quantitative real-time RT-PCR as described in Materials and Methods. B, CO release under different treatments at 12 h. Values were the mean ± SE of three independent experiments. Bars denoted by the different letter differed significantly at *P<*0.05 according to Duncan's multiple range tests.

We analyzed the expression of *HO-1* mRNA in wheat seedling leaves in response to the transition from dark to light at different times. Quantitative real-time RT-PCR revealed that seedling leaves of wheat plants transferred from dark to light (D→L) with dark- and light-grown controls (D→D, and L→L) brought about the highest induction of *HO-1* gene (Fig. 3A). For example, the results shown that *HO-1* mRNA level was increased more than 3 times after transition (12 h) respect to control values, while 1.5 time enhancement was observed at 6 h of transition. This fact indicated that *HO-1* gene response occurred in a time-dependent manner. On the other hand, treatment either with dark or light for 12 h produced the slightly decreased expression pattern of *HO-1* (Fig. 3A).

To assess whether the aforementioned different mRNA levels reflected changes in HO activities, enzyme activity determination was performed. As shown in Table 1, the observed differences in transcripts were also positively correlated with enzyme activities. Interestingly, it is noteworthy that, as expected, treatment with ZnPPIX in seedling leaves of wheat plants transferred from dark to light produced the significantly decrease of HO activity (*P*<0.05). Moreover, the changes of CO content under different treatments were in conformity with HO activities (Fig. 3B).

**Table 1:** Time course of changes in HO activities (U mg protein^−1^) during transition from dark to light.

Treatment (h)	L→L	D→D	D→L	D→L+ZnPPIX
0	0.35±0.05a	0.23±0.02a	0.24±0.07d	0.26±0.04a
3	0.32±0.04a	0.21±0.01a	0.51±0.07c	0.05±0.02b
6	0.31±0.09a	0.19±0.04a	0.85±0.04b	0.05±0.00b
12	0.30±0.05a	0.18±0.07a	1.29±0.01a	0.04±0.01b

μmmol m^−2^s^−1^) or dark (D) for 5 d (25°C). Afterwards, seedlings were cultured in the Hoagland solution with or without 100 μM HO-1 inhibitor ZnPPIX treatment, in the light (L) or dark (D) for another 12 h (25°C). Before starting the experiments, 14-day-old wheat seedlings cultured in the Hoagland solution were kept in the light (L, 300

± SE of three different experiments with at least three replicated measurements. Different letters within columns indicate significant differences (*P*<0.05) according to Duncan's multiple range test. Values are means

### HO activities and *HO-1* transcripts were increased in response to SNP, hematin, and CO aqueous solution in etiolated wheat seedling leaves

**Figure 4:**
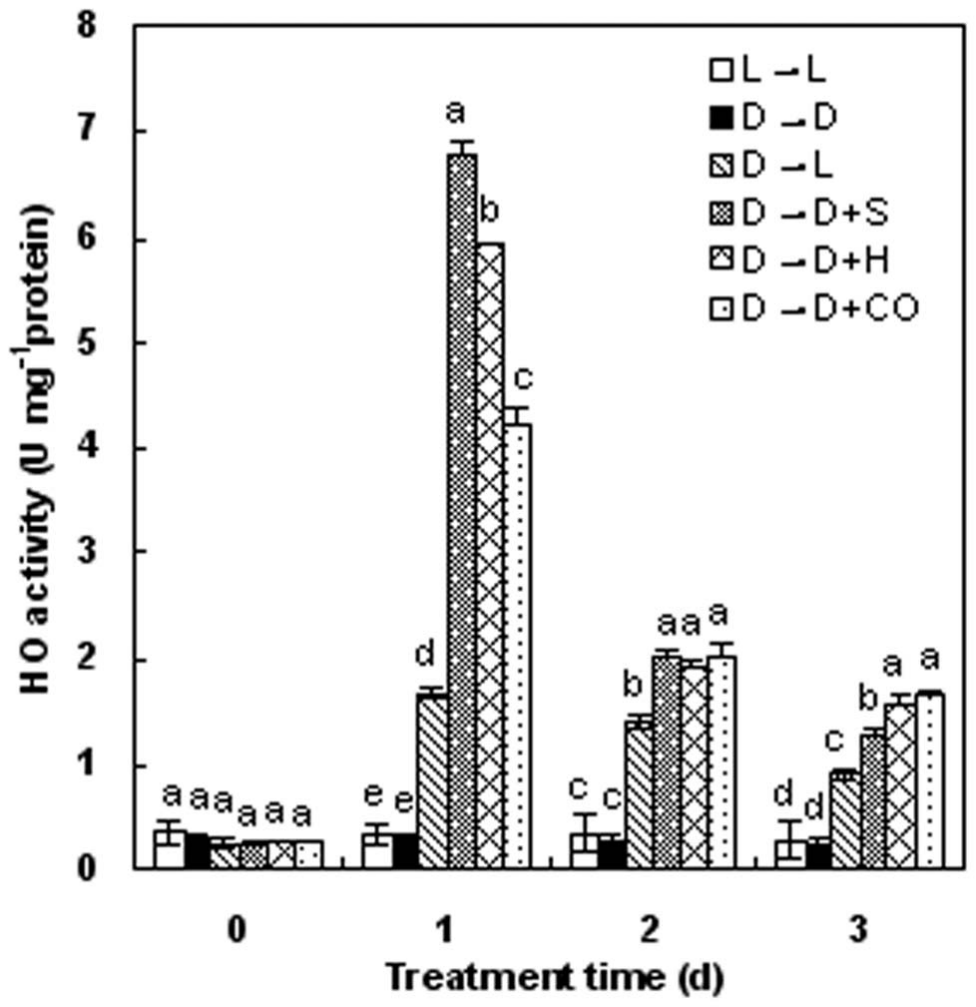
Effects of NO donor SNP, hematin and CO-saturated aqueous solution on HO activities in wheat seedling leaves after dark pretreatment. Before starting the experiments, 14-day-old wheat seedlings cultured in the Hoagland solution were kept in the light (L, 300 μmmol m^−2^s^−1^) or dark (D) for 5 days. Afterwards, seedlings were cultured in the Hoagland solution without (D→D) or with SNP (S, 100 μM), HO-1 inducer hematin (H, 10 μM), 1.0% CO-saturated aqueous solution (CO) in completely darkness for another 3 days. Values were the mean ± SE for at least three independent experiments. Bars denoted by the same letter did not significantly differ at *P<*0.05 according to Duncan's multiple range tests.

**Figure 5:**
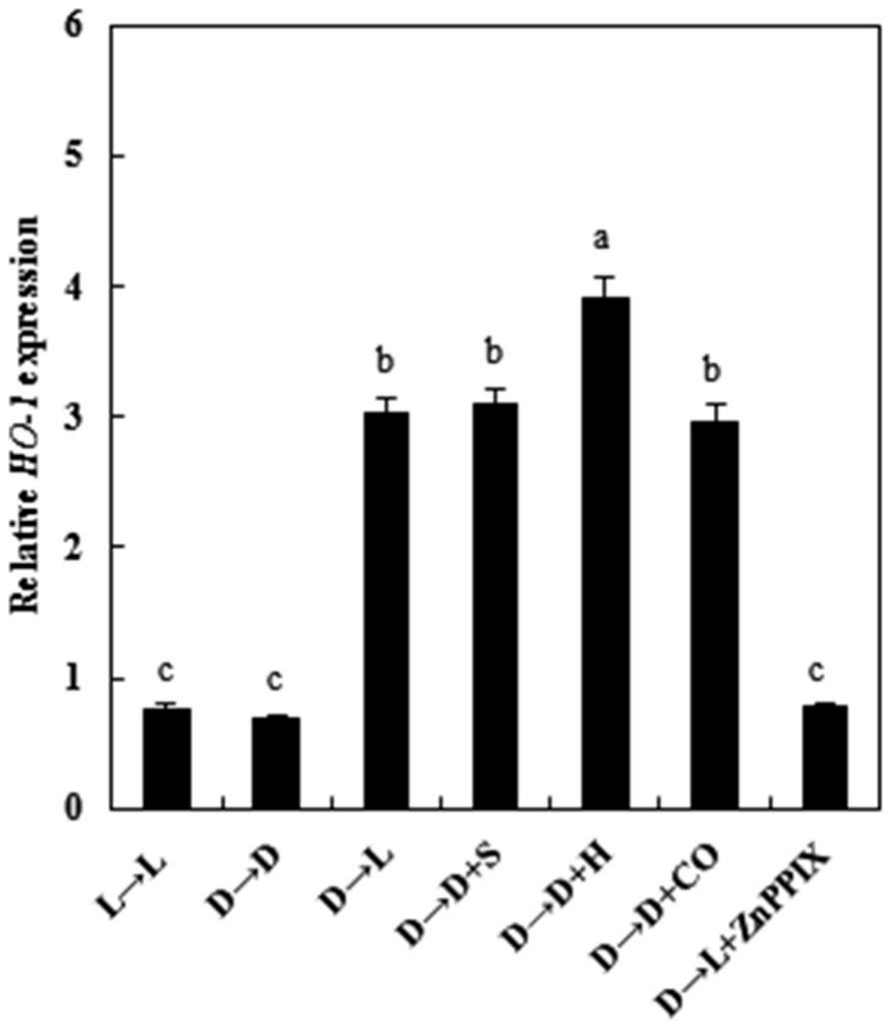
HO-1 inducer hematin, exogenous CO and light treatments induce *HO-1* gene expression in wheat seedling leaves after dark pretreatment. Before starting the experiments, 14-day-old wheat seedlings cultured in the Hoagland solution were kept in the light (L, 300 μmmol m^−2^s^−1^) or dark (D) for 5 days. Afterwards, seedlings were cultured in the Hoagland solution without (D→D) or with HO-1 inducer hematin (H, 10 μM), and 1.0% CO-saturated aqueous solution (CO) for another 12 h. *HO-1* mRNA expression was analyzed by quantitative real-time RT-PCR as described in Materials and Methods. Three independent experiments were performed, bars denoted by the same letter did not significantly differ at *P<*0.05 according to Duncan's multiple range tests.

The light-like effect of applying HO-1 inducer hematin or CO aqueous solution to dark-grown samples first led us to investigate whether changes in endogenous HO activities and *HO-1* transcripts occurred in response to hematin or CO aqueous solution, as well as these actions of light (Fig. 3, Table 1), or a well known NO donor sodium nitroprusside (SNP). In our experimental conditions, 10 μM hematin (H), or 1.0% CO aqueous solution (CO), applied to dark-grown wheat seedlings, induced rapid enhancement of HO activity (Fig. 4). The time course test shown that in comparison with dark-grown controls (D→D), a rapidly maximum response was discovered as early as 1 day after different treatments (D→D+S, D→D+H, and D→D+CO), followed by a gradual decrease until the third day (Fig. 4). A weaker increased level of HO activities after treatment in the transition from dark to light (D→L) was also observed (Fig. 4). Meanwhile, changes in *HO-1* transcripts (Fig. 5) were found to be similar to those for HO activities under different treatments. We also noted that the induction of HO expression elicited by hematin apparently preceded light-induced de-etiolation.

### The application of L-NAME and cPTIO blocked the restoration of chlorophyll contents in etiolated wheat seedling leaves induced by SNP, hematin, and CO aqueous solution

**Figure 6:**
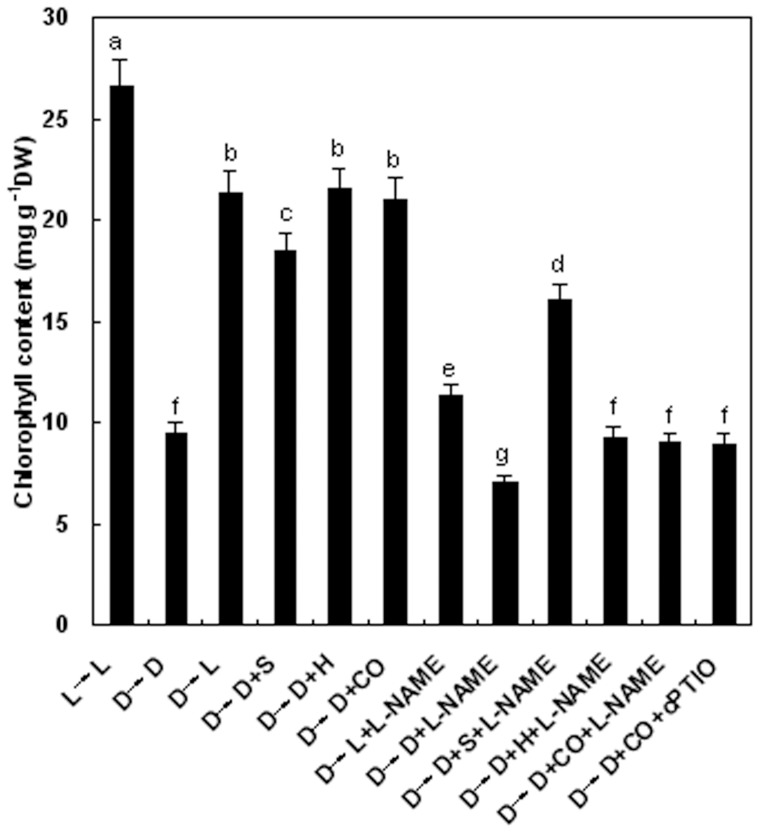
SNP, hematin, CO-saturated aqueous solution, CO scavenger hemoglobin, mammalian NOS-like inhibitor L-NAME, and the NO specific scavenger cPTIO differentially influence the chlorophyll content in etiolated wheat seedling leaves after dark pretreatment. Before starting the experiments, 14-day-old wheat seedlings cultured in the Hoagland solution were kept in the light (L, 300 μmmol m^−2^s^−1^) or dark (D) for 5 days. Afterwards, seedlings were cultured in the Hoagland solution without or with 100 μΜ SNP (S), 10 μM HO-1 inducer hematin (H), 1.0% CO aqueous solution (CO), 0.1 g L^−1^ Hb, 200 μM L-NAME, 100 μM cPTIO, or the above combination treatments, in the light (L) or dark (D) for another 3 days. Values were the mean ± SE for at least three independent experiments. Bars denoted by the same letter did not significantly differ at *P*<0.05 according to Duncan's multiple range tests.

The observation that de-etiolation of wheat seedling leaves displayed similar sensitivity to light, SNP, hematin, and CO aqueous solution prompted us to examine the effects of the inhibitor of mammalian NOS-like protein (L-NAME) and a specific NO scavenger cPTIO on the changes of chlorophyll contents in etiolated wheat seedling leaves with or without SNP, hematin, and CO aqueous solution. As shown in Fig. 6, similar to the dark-induced decay of chlorophyll, the ameliorating effects of SNP (S), hematin (H), and CO aqueous solution (CO) on etiolation was significantly blocked by L-NAME or cPTIO, respectively. Further, the negative effects of L-NAME and cPTIO on light-induced de-etiolation were observed. These results suggested that endogenous NO is likely to be involved in de-etiolation elicited by light, hematin and CO aqueous solution, and that mammalian NOS-like enzyme-mediated NO production plays an important role in above process.

### NO production was increased in response to SNP, hematin, and CO aqueous solution in etiolated wheat seedling leaves

**Figure 7:**
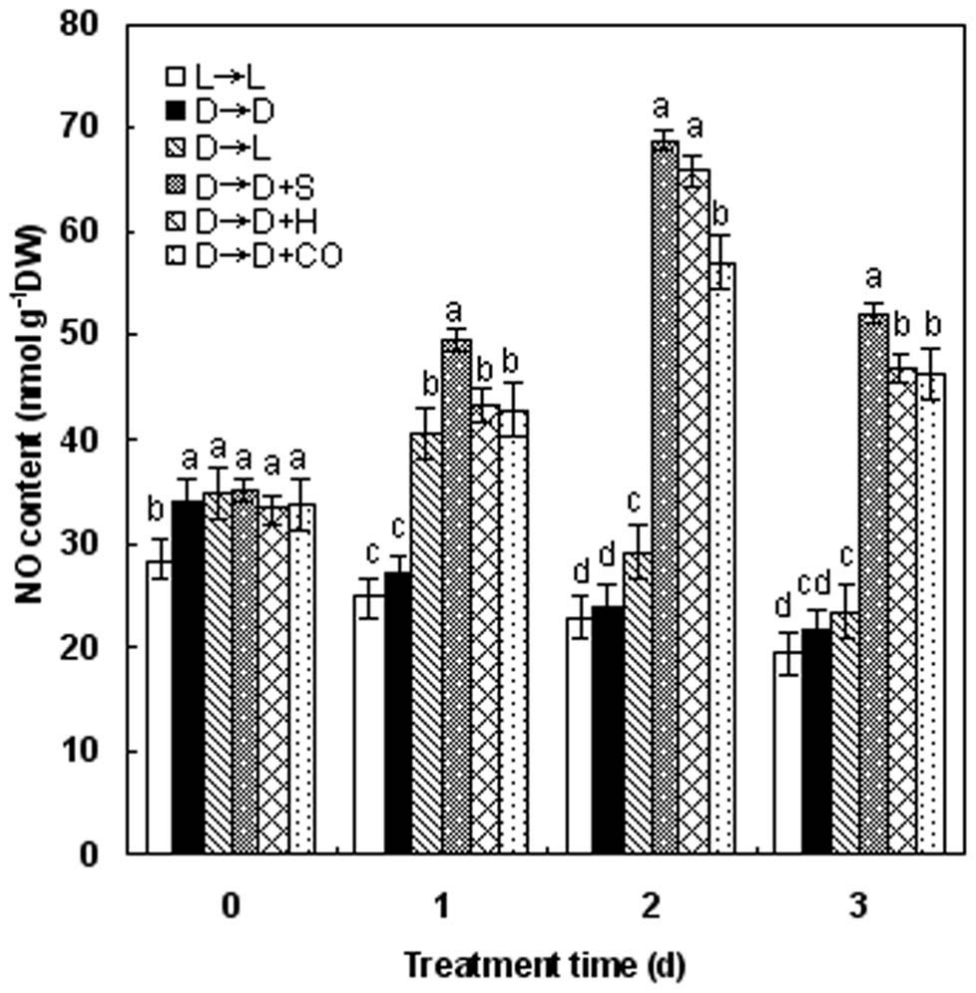
Time course of endogenous nitric oxide (NO) generation induced by SNP, hematin and CO aqueous solution in wheat seedling leaves. Before starting the experiments, 14-day-old wheat seedlings cultured in the Hoagland solution were kept in the light (L, 300 μmmol m^−2^s^−1^) or dark (D) for 5 days (25°C). Afterwards, seedlings were cultured in the Hoagland solution without or with 100 μΜ SNP (S), 10 μM HO-1 inducer hematin (H), and 1.0% CO aqueous solution (CO), in the light (L) or dark (D) for another 3 days. NO contents were detected by using Greiss reagent. Values were the mean ± SE for at least three independent experiments. Bars denoted by the same letter did not significantly differ at *P*<0.05 according to Duncan's multiple range tests.

**Figure 8:**
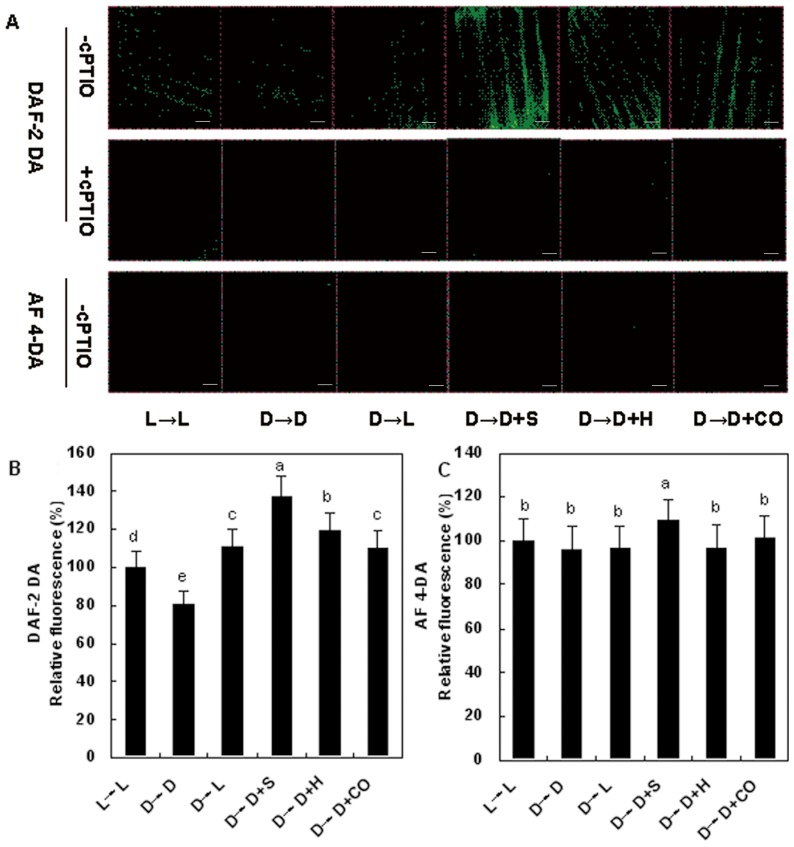
NO accumulation in etiolated distilled water was taken as control. The distribution of nitric oxide (NO) in wheat seedling leaves induced by SNP, hematin, and CO aqueous solution in wheat seedling leaves. Before starting the experiments, 14-day-old wheat seedlings cultured in the Hoagland solution were kept in the light (L, 300 μmmol m^−2^s^−1^) or dark (D) for 5 days. Afterwards, seedlings were cultured in the Hoagland solution without or with 100 μΜ SNP (S), 10 μM HO-1 inducer hematin (H), and 1.0% CO aqueous solution (CO), in the light (L) or dark (D) for another 2 days. A, The NO distribution was detected by fluorescence probe DAF-2 DA and negative probe AF 4-DA 2 days after different treatments under fluorescence microscopy (TCS-SP2 confocal laser scanning microscope; Leica Lasertechnik GmbH). B, Mean relative DAF-2 DA and AF 4-DA fluorescence densities corresponding to samples without cPTIO treatment was given. Values were the mean ± SE for at least three independent experiments. Bars denoted by the same letter did not significantly differ at *P*<0.05 according to Duncan's multiple range tests. Bars = 20 μm.

To further confirm whether de-etiolation elicited by light, hematin, and CO aqueous solution is related to endogenous NO, Greiss reagent method (Fig. 7) or a fluorescence method with or without the specific fluorescent probe 4,5-diaminofluorescein diacetate (DAF-2 DA), the negative probe 4-amino fluorescein diacetate (AF 4-DA), and NO scavenger cPTIO (Fig. 8), was used to evaluate the changes of NO signal, respectively. As expected [31], in comparison with the dark-grown control samples (D→D), the time course experiments for additional three days illustrated that a burst of endogenous NO production appeared on the second day with light, SNP, hematin, and CO treatments, respectively, and then followed by a gradual decrease till the third day (Fig. 7).

Furthermore, a slight decrease of DAF-2 DA-dependent green fluorescence was firstly observed in the dark-grown sample (D→D, 2 d) respect to the light-grown control (L→L). The green fluorescence was increased by the addition of the NO donor SNP and reduced by the specific scavenger cPTIO, respectively, further confirming that DAF-2 DA-dependent green fluorescence is associated with endogenous NO concentration. As expected (Fig. 7), an increase in endogenous NO production in seedling leaves was found upon exposure to light, SNP, hematin, and CO, as demonstrated by 37.4, 69.2, 47.7 and 36.6% induction of fluorescence intensity following 2 days. cPTIO, a specific scavenger of NO, also inhibited above inductions. Moreover, similar increase in fluorescence was not apparently observed when AF 4-DA, the negative control probe for DAF-2 DA, was applied, respectively. Together, these results reveal that an induction of endogenous NO production in the seedling leaves might be a critical event in above effects elicited by light, SNP, hematin, and CO aqueous solution during de-etiolation process.

### Application of SNP, hematin and CO saturated aqueous solution induced the accumulation of Pfr in etiolated wheat seedling leaves

**Figure 9:**
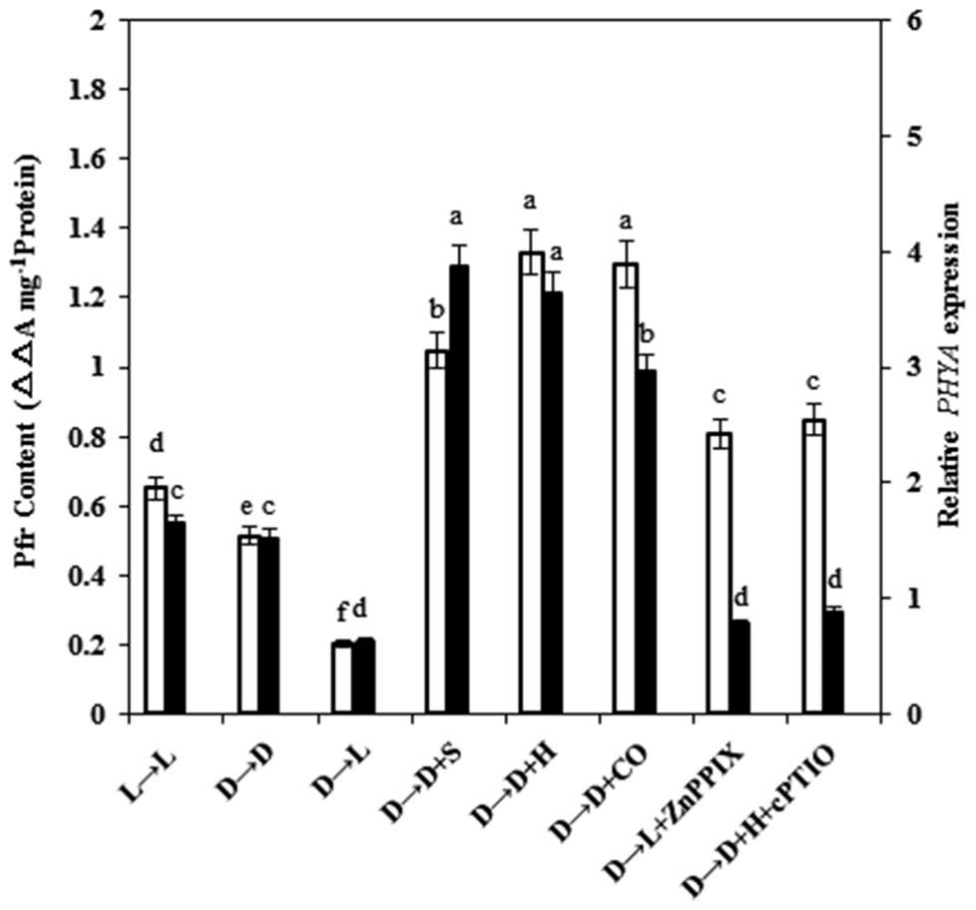
Effects of treatment with SNP, hematin, CO-saturated aqueous solution, ZnPPIX, and cPTIO on Pfr contents (3 d) and expression profiles of *PHYA* expression (2 d). 14-day-old wheat seedlings were grown for 5 days at 25°C in continuous darkness (D) or light (L) at 300 μmmol m^−2^s^−1^ before either being transferred into light or left in continuous darkness without or with 100 μM SNP, 10 μM hematin, 1.0% CO aqueous solution, 100 μM HO-1 specific inhibitor ZnPPIX, and 100 μM cPTIO, or their combination treatments, for another 3 days. *PHYA* mRNA expression was analyzed by quantitative real-time RT-PCR as described in Materials and Methods. Values were the mean ± SE for at least three independent experiments (n = 20). Bars denoted by the same letter did not significantly differ at *P<*0.05 according to Duncan's multiple tests.

Phytochromes are molecular light switches that regulate various aspects of plant growth and development. Phytochromes exist in two photointerconvertible forms, Pr and Pfr. Pfr is thought to be the active form of phytochrome, and can be converted back to Pr by far-red light. Therefore, we further detected the relative amount of Pfr to investigate whether HO/CO involving in light-mediated signals in plants depend on photoreceptors. Results shown in Fig. 9 illustrated that Pfr content in dark-grown plant (D→D) was decreased with respect to the light-grown control (L→L) (*P*<0.05). By comparison, the dark-induced decrease of Pfr level was markedly recovered by SNP, hematin, and CO aqueous solution treatment for additional 3 days. On the other hand, ZnPPIX or cPTIO significantly blocked hematin-induced effects. Meanwhile, dark-light transition (D→L) even led to the more serious decrease of Pfr content.

It is previously reported that in dark-grown seedlings, PHYA is the most abundant phytochrome, which is responsible for de-etiolation under far-red light [8]. In the following test, we analyzed the expression profile of *PHYA* using quantitative real-time RT-PCR and discovered that SNP, hematin and 1.0% CO saturated solution all were able to induce *PHYA* gene expression as well as dark treatment (Fig. 9A). However, D→L treatment also exhibited no obvious positive effect.

## Discussion

In animal research, HOs have been widely studied and well understood. Recently, an increasing number of researches concentrate on its function in plants. It is an important effecter of growth, development and defense response, but there were few reports about the involvement of HO/CO in formation of photosynthetic performance. In our experiment, we discovered that when etiolated wheat seedling induced by darkness, were transferred to light for greening, HO activities, *HO-1* transcript, and CO release were obviously up-regulated (Fig. 3), which were consistent with the increase of chlorophyll content (Fig. 1). However, addition of ZnPPIX blocks this inducible effect. It is suggested that HO/CO system might participate in light-induced wheat seedling de-etiolation.

An earlier study [32] indicated that when treated with hematin, NaCl-induced chlorophyll decay of wheat seedling leaves was evidently reduced. Our results also confirmed that application of hematin, a HO-1 inducer or CO donor, could impair the reduction of chlorophyll contents in entirely darkness, but treatment with Hb (a CO scavenger) arrested this positive effects during the greening process (Fig. 1). The block action of Hb suggested that endogenous CO, may possibly from HO, be responsible for wheat seedling de-etiolation. Meanwhile, lower concentration of CO aqueous solution acted as the same function on restoring the decrease of chlorophyll contents, which also confirmed above hypothesis. Besides, hematin (H), or 1.0% CO aqueous solution (CO), could enhance HO activity in dark-grown wheat seedlings (Fig. 4), which suggested that hematin, acting as the substrate of endogenous HO and exogenous lower concentration of CO aqueous solution might regulate wheat seedling de-etiolation in absence of light through activating endogenous HO/CO system.

Zhang et al. [33] reported that endogenous NO participated in light-inducing greening of etiolated barley seedlings. In this study, based on the relationship between HO/CO and NO shown previously in animals and recently in plants, we investigated the putative role of NO in light, hematin, and CO-induced responses by determining NO *in vivo* using fluorescence probe as well as the usage of chemicals that can manipulate the NO concentrations during greening process. Our results indicated that hematin and CO could induce the production of endogenous NO and its peak value appeared on the second day, which lag behind the increase of HO activity. Application of NOS inhibitor L-NAME (200 μM) and NO scavenger cPTIO could block the increase of chlorophyll content induced by hematin and 1.0% saturated CO aqueous solution in etiolated wheat seedlings, which suggested that NO might intermediate wheat seedling de-etiolation aroused by light or hematin/CO. Intriguingly, we also discovered that SNP treatment could also induce the elevation of HO activities in time-dependent manner in wheat seedling greening process, which was indicated by Noriega et al. [4] in soybean leaves. Based on these results, it is deduced that there exists the possible link between HO/CO and NO in wheat seedling de-etiolation in absence or presence of light.

Phytochromes are traditionally known as biliprotein photoreceptors in plants and PHYA is predominantly responsible for de-etiolation. To investigate the role of phytochrome in light or hematin/CO induced de-etiolation, we analyzed the changes of Pfr content and discovered that the amount of Pfr and *PHYA* expression were more substantive in hematin/CO treated wheat seedlings in completely darkness (Fig. 9). Furthermore, continuous dark treatment (D→D) also exhibited the same inducible effects. However, when wheat seedlings transferred from dark to light, both Pfr content and *PHYA* transcripts were decreased. From these data we could presume that hematin and CO accomplish wheat seedling de-etiolation process by increasing phytochrome expression through the following way: hematin and exogenous CO might activate endogenous HO activities to synthesize the precursors of the chromophores for the plant light-harvesting photobiliproteins [34], [35]; another offspring, endogenous CO up-regulated the expression of phytochrome apoprotein genes. Afterwards, both of them linked together through a thiol-ether bond to form integrated phytochrome which could regulate chlorophyll accumulation through influencing the formation of 5-aminolevulinate [36]. SNP, a potent NO donor have more obvious function on increasing the content of Pfr than that hematin and CO did; whereas, adding cPTIO led to the decrease. Therefore, it is speculated that phytochrome might mediate the HO/CO signal transduction and act as a downstream regulator of HO/CO and NO. The mechanism of HO/CO in regulating wheat seedlings greening in continuous darkness might be different from light inducible effects. Chlorophyll and heme are both synthesized in the plastid from ALA and share a common pathway between ALA and Proto IX [37]. In the dark chlorophyll synthesis proceeds only as far as protochlorophyllide because the enzyme protochlorophyllide oxidoreductase (POR) has an absolute requirement for light. That etiolated seedlings transferred from dark to light activate the activity of POR, which finally leads to the chlorophyll accumulation. In the absence of light, the increase of HO activities accelerated the degradation of endogenous heme and consequently speeded up their common metabolic pathway, as a result, improving chlorophyll synthesis.

To our knowledge, the present study provided the first description of endogenous HO/CO and NO in wheat seedling de-etiolation. Both hematin and SNP displayed effects on up-regulation of endogenous HO/CO system and NO releasing. The mutual induction effects suggested that there might be an inseparable relationship between HO/CO and NO during the greening process of wheat seedlings. Moreover, HO/CO system and NO might achieve the physiological function described above by modulating phytochrome transformation and synthesis.

## Materials and Methods

### Plant Materials and growth condition

Seeds of wheat (*Triticum aestivum* L.) were carefully selected and sterilized with 5% NaClO for 15 minutes, then washed extensively with distilled water. These seeds firstly were germinated in plastic box wetted by distilled water at 28°C in growth chamber in the darkness. After about 24 hours, all seeds burgeoning were moved to floating planks for about 3 days. Then the thriving seedlings were transferred to the plastic beakers and kept in growth chamber (12-h light period, 25°C, humidity 50%±4%; 12-h dark period, 18°C, humidity 56%±5%, MGC-300B, Shanghai Yiheng Technology Co., Ltd., China) with modified Hoagland solution [38] containing 3 mM KNO_3_, 1 mM NH_4_H_2_PO_4_, 0.5 mM MgSO_4_, 5.5 mM Ca(NO_3_)_2_, 50 mg of Fe-EDTA per liter (10% iron), 25 μM KCl, 12.5 μM H_3_BO_3_, 1 μM MnSO_4_, 1 μM ZnSO_4_, 0.25 μM CuSO_4_, and 2 μM H_2_MoO_4_ for nearly 14 days. The irradiance was approximately 300 μmol m^−2^s^−1^ provided by fluorescent lamps. The culture solution was renewed every other day until two fully expanded leaves appeared.

### Chemicals

All chemicals were purchased from Sigma (St Louis, MO, USA) unless otherwise stated. Sodium nitroprusside (SNP) was used at 0.1 mM as an NO donor. Hematin (H, C_34_H_33_N_4_O_5_Fe), was used as HO-1 inducer at concentrations of 1.0, 10, 100 μM. Also, Hemoglobin (Hb), obtained from Shanghai Boao Ltd., China, was chosen as the scavenger of CO/NO at the concentration of 0.1gL^−1^ [30], [39]. The compound 2-(4-carboxyphenyl)-4,4,5,5- tetramethylimidazoline-1-oxyl-3-oxide potassium salt (cPTIO) was used as a specific NO scavenger. *N^G^*-nitro-L-arginine methylester hydrochloride (L-NAME) was used as the inhibitor of nitric oxide synthase (NOS) and the compound zinc protoporphyrin (ZnPPIX) was used as a potent inhibitor of HO-1. NO specific fluorophore 4,5-diaminofluorescein diacetate (DAF-2 DA) and AF 4-DA (4-amino fluorescein diacetate) was purchased from Calbiochem (San Diego, CA, USA), used at a final concentration of 10 μM [40].

### CO-saturated aqueous solution preparation

CO gas was prepared by heating concentrated sulfuric acid (H_2_SO_4_) with formic acid (HCOOH) at the speed of 3–5 seconds per drop. In our experiment, CO-saturated aqueous solution was freshly obtained by bubbling above CO gas gently through a glass tube into 300 ml of above Hoagland solution for about 30 min, a duration time long enough to make the solution saturated with CO. Then the saturated stock solution (100% of saturation) was immediately diluted with fresh prepared Hoagland solution to the concentration the experiment required (0.1, 1.0, 10 and 50% of saturation).

### Treatments

Wheat seedlings were grown in growth chamber until the second leaves were fully expanded nearly for 14 days. The further experimental design thus consisted of the following treatment groups: L→L: stands for wheat seedlings were grown in normal condition. Other seedlings were kept in complete darkness for five days. After that, they were treated with different concentrations of hematin (1.0, 10, and 100 μΜ) and CO-saturated aqueous solution (0.1, 1.0, 10, and 50%) for a few additional days in the dark (abbreviated to D→D+H1.0, 10, and 100 or D→D+CO0.1%, 1.0%, 10%, and 50%). Among them, 10 μΜ hematin and 1.0% CO-saturated aqueous solution were chosen as the suitable treatment because of its better positive results, termed as D→D+H and D→D+CO in the following experiments. 100 μΜ SNP treatment was brought into comparison (abbreviated to D→D+S). D→D stands for these five-day dark-treated seedlings were continuous kept in the darkness. When these etiolated seedlings were transferred to light again (300 μmol m^−2^s^−1^), it was abbreviated to D→L. NOS inhibitor L-NAME (200 μM), NO scavenger cPTIO (100 μΜ), NO/CO scavenger Hb (0.1 gL^−1^), and HO-1 inhibitor ZnPPIX (100 μΜ) were used respectively. All groups were arranged in plastic beakers with at least three replicates. Additionally, all the containers were separated to avoid the CO gas escaping or any possible CO gas interference, and above solutions were renewed each day. All tests were carried out at least three independent sets of experiments with similar results.

### Chlorophyll content determination

Chlorophyll quantification was performed by the method described by Sa et al. [41].

### Heme oxygenase preparation and activity determination

Preparation of crude membrane fractions and detection of HO activity were determined by the method of Liu et al. [42]. HO activity was calculated by measuring the formation of biliverdin-IXα (BV). The concentration of BV was estimated using a molar absorption coefficient at 650 nm of 6.25 mM^−1^cm^−1^ in 0.1 M HEPES-NaOH buffer (pH 7.2). One unit of activity (U) was calculated by taking the quantity of the enzyme to produce 1 nmol BV at 37°C per 30 min.s

### Phytochrome content determination

Phytochrome content was determined by the method of Lane et al. [43]. The phytochrome in wheat seedlings was assayed with a dual-wavelength difference photometer. The instrument measured the optical-density difference, ΔOD, between 730 and 800 mp, rather than the difference between 660 and 730 my, in order to eliminate the optical-density changes due to protochlorophyll transformations. The relative amounts of Pfr could be determined. After a given treatment, measurement of the sample would give a certain ΔOD reading, R1, on the instrument. The sample would then be irradiated with the actinic source of far-red light to convert any Pfr present to Pr and decrease the ΔOD reading to R2. The amount of Pfr would be equal to k (R1-R2), where k is a constant of proportionality. The difference between ΔOD readings is referred to as Δ(ΔOD).

### Protein content determination

Protein concentration was determined by the method of Bradford [44] using BSA as the standard.

### Real-time RT-PCR analysis

Real-time quantification RT-PCR reactions were performed in a step one plus real-time PCR system (Applied Biosystems, America) using the SYBR® Premix Ex TaqTM (TaKaRa Bio Inc., Dalian, China) according to the manufacturer's instructions. The PCR reaction was performed using the following primers: for *HO-1* (GenBank ID: HM014348), (F: 5′-AATACTGGGTTGGAGAGA-3′ and R: 5′-AGAAGTGGCAAATAAATG-3′); and for *Actin* (GenBank ID: AB181991), (F: 5′-TCTGGTGATGGTGTGAGC-3′ and R: 5′-CGGTTGTTGTGAGGGAGT-3′). For *PHYA* (GenBank ID: AJ313099) (F: 5′- TTACAGGGTATGATAGGG -3′ and R: 5′- AGTGGCTGGATAGTGC -3′). Gene-specific primers were designed with the software tool Primer Express (Applied Biosystems, Foster city, CA, USA). All reactions were set up in triplicate. Relative expression levels are presented as values relative to corresponding control sample at the indicated times, after normalization to *Actin* transcript levels.

### NO content determination by using Greiss reagent

NO content from wheat leaves was determined using the method described by Zhou et al. [45]. Absorbance was assayed at 540 nm; NO content was calculated by comparison to a standard curve of NaNO_2_.

### Imaging of endogenous NO by LSCM

In our experiment, 2.0–2.5 mm transversal sections from the center of leaves, was further treated in the presence or absence of 1 mM cPTIO for 50 min, and then incubated with the specific fluorescent probe DAF-2 DA in 20 mM BES-KCL buffer (pH6.2) in darkness for 1 h. The extra probes were removed by washing the sections at 20 mM BES-KCL buffer three times for 15 min and then images were taken using a TCS-SP2 laser scanning confocal microscope (Leica lasertechnik Gmbh, Heidelberg, exciting with the 488 nm, and emission using 490–530 nm for NO analysis). The negative probe AF 4-DA was also used as control. Experiments were repeated six times and similar results obtained. All manipulations were performed at 25°C±1°C.

### CO content determination

The determination of CO content by gas chromatography and mass spectrometry (GC/MS) were carried out according to the method described by our previous report [42].

### Statistical analyses

Each experiment was repeated at least three times with similar results. Values were expressed as means ± SE at least three replicates obtained from three independent experiments. Different measurements were subjected to analysis of variance (ANOVA) using SPSS with significances of *P*<0.05 and *P*<0.01, respectively.
